# Sexual Inactivity in Methadone Maintenance Treatment Patients

**DOI:** 10.3390/ijerph17061993

**Published:** 2020-03-18

**Authors:** Fitri Fareez Ramli, Tg Mohd Ikhwan Tg Abu Bakar Sidik, Isa Naina Mohamed

**Affiliations:** 1 Department of Pharmacology, Faculty of Medicine, Universiti Kebangsaan Malaysia, Kuala Lumpur 56000, Malaysia; fitrifareez@ppukm.ukm.edu.my; 2 Pharmacoepidemiology and Drug Safety Unit, Department of Pharmacology, Faculty of Medicine, Universiti Kebangsaan Malaysia, Kuala Lumpur 56000, Malaysia; tgikhwancrc@gmail.com

**Keywords:** sexual inactivity, methadone, opioid use disorder

## Abstract

Sexual dysfunction has been extensively studied in methadone maintenance treatment (MMT) patients. However, little data is available regarding sexual inactivity in the MMT patient population. The objectives of this study were to determine the prevalence and putative risk factors for sexual inactivity in the MMT patient population. This cross-sectional study involved 25–71 year old MMT patients recruited from six methadone clinics. Two hundred and seventy-one patients were interviewed for demographic characteristics, comorbidities, concurrent medications used, and sexual activity. The prevalence of sexual inactivity in the MMT population was found to be 47.6%. Increasing age (*p* < 0.01) and being single/divorced (*p* < 0.01) were significantly associated with sexual inactivity. In subgroup analysis, increasing age was significantly associated with sexual inactivity in both single/divorced (*p* < 0.05) and married (*p* < 0.05) subgroups, while unemployment (*p* < 0.05) was only significantly associated with sexual inactivity in the earlier subgroup. Our results suggest that sexual inactivity is common in the MMT patient population. The putative risk factors are related to biological and sociocultural factors. Having specific comorbidities or being on certain medications were not correlated with sexual inactivity in the MMT population. Routine assessment of sexual problems is essential, and proper management should be performed for MMT patients.

## 1. Introduction

Methadone is a strong opioid agonist. Its long duration of action, reserve for tolerance effect [1], and its ability to control illicit opioid use [2], makes it favorable as a replacement therapy. Methadone is indicated for opioid use disorder to manage opioid addiction and has been proven to reduce harmful drug-seeking behavior [2]. In Malaysia, the methadone maintenance treatment (MMT) program was introduced back in 2005 as part of the harm reduction program for people who inject drugs (PWID) [3,4]. Even though the number of opioid users had decreased by half in 2017, the number of patients on MMT remained high [3]. According to the Malaysian national key population estimates report in 2018, it was estimated that more than 30,000 PWID were on the MMT program [3]. MMT patients deserve significant attention, as methadone is associated with various adverse effects, such as sexual dysfunction [5,6].

Sexual function is one of the vital needs in life. Disturbance of sexual function can affect quality of life [7,8] and relationships [9]. In the male general population, sexual inactivity ranges between 15.9% to 17.6% [10,11]. The prevalence of sexual inactivity is higher in people with comorbidities such as diabetes, hypertension, psychiatry conditions, and human immunodeficiency virus (HIV), than the general population [12,13,14]. Sexual activity tends to reduce with age [15]. Factors associated with sexual inactivity are multifactorial and include increasing age, relationship status, educational status, and employment status [10,16,17].

Various studies have reported the association between MMT and sexual dysfunction [5,6]. However, little is known about sexual inactivity in this population. To date, only one study has evaluated sexual activity in opioid replacement treatment patients on both buprenorphine replacement treatment (BMT) and MMT. This study found that the proportion of sexual inactivity in MMT patients was four times higher than in the BMT group [18]. However, the correlates for sexual inactivity were not determined in each specific treatment group. To the best of our knowledge, as yet there has been no study conducted evaluating sexual activity, specifically in the MMT population. This study aimed to determine the prevalence of sexual inactivity and putative risk factors associated with sexual inactivity in the MMT population.

## 2. Materials and Methods

The design of the study was cross-sectional. The study was conducted in six primary health care clinics located in Kuala Lumpur, Malaysia, between January to April 2019. Primary healthcare is the main center for MMT in Malaysia. The distribution of patients taking MMT in primary healthcare is 77% [2]. All MMT patients attending these clinics were invited to participate in the study. No compensation was given to the patients involved in this study. Inclusion criteria were that participants were male, aged at least 18 years old, and had been on methadone treatment for at least six months. Based on Ali et al. [2], the Malaysian female population on MMT was less than 1%. Given females represent a very small percentage of the MMT population, we did not include females in the present study. Ethical approval was obtained from the Research Ethics Committee, Universiti Kebangsaan Malaysia (JEP-2018-622), and the Medical Research & Ethics Committee, Ministry of Health, Malaysia (NMRR-18-2586-42421). Written informed consent was acquired from all patients for participation in this study.

### 2.1. Data Collection

Demographic data were obtained from the patients. Data recorded were age, ethnicity, marital status, education level, and employment status. Clinical data such as hepatitis (both B and C), HIV, hypertension, diabetes mellitus, psychiatric disorder, and the use of medications for the treatment of those conditions were inquired from the patient and traced from medical records. The dose and duration of methadone treatment were also recorded. 

### 2.2. Assessment of Sexual Inactivity

Patients were asked about any sexual activity within the past six months. Sexual inactivity is defined as not having any sexual activity involving a partner for the past six months before the interview.

### 2.3. Statistical Analysis

All analyses were performed using the Statistical Package for Social Science (IBM Corp. Released 2013 IBM SPSS Statistics for Windows, Version 22.0. Armonk, NY: IBM Corp.). Continuous variables are described using summary statistics (mean and standard deviation) for normally distributed variables. If the distribution was not normal, median and inter-quartile range (IQR) are reported. Categorical (nominal/ordinal) variables are described by the frequency with percentage.

Demographic, treatment, and clinical factors associated with sexual inactivity were identified using univariable analysis. Independent *t*-test was used for the univariable analysis of continuous variables with normal distribution, and the Mann-Whitney test was used for variables with skewed distribution. Chi-square test or Fisher’s exact test (when minimum expected count is less than five) were used for the univariable analysis of categorical variables. The crude odds ratio (OR) was calculated with a 95% confidence interval (CI) for all risk factors.

The associations of each potential putative risk factors were examined through multivariable analysis. Using the purposeful selection method [19], the significant factors were identified by fitting all independent factors with a *p*-value of less than 0.25 in univariable analysis into multiple logistic regression. Adjusted ORs with 95% CI were reported, and a p-value of less than 0.05 was considered statistically significant with two-sided test.

## 3. Results

335 out of the 433 active MMT patients were recruited from six primary health care clinics. Out of this sample, 18 patients refused to participate. Twenty-nine patients were excluded because the duration of MMT treatment was less than six months. A total of 271 patients were interviewed for demographic, clinical data, and sexual activity.

### 3.1. Demographic Characteristics

The mean age of our population was 48.8 years (SD ± 9.7). The population age ranged from 25–71 years old. Our study population was 68.6% Malay, 46.1% married, 69.6% employed, and 76.0% had a secondary school education level. The mean and standard deviation of methadone dose was 59.7 mg (SD ± 32.5). The median of methadone duration was 36.0 months (IQR 16.0–63.0). Half of our population had hepatitis C (51.9%), 16.6% had hypertension, 5.9% had HIV, 4.4% had diabetes mellitus, 4.1% reported had psychiatry disorder, and only 3.3% had hepatitis B. In terms of medications, 1.5% of patients with hepatitis were on medication, 4.1% on highly active antiretroviral therapy (HAART), 14.0% on anti-hypertensive treatment, 3.3% on anti-diabetic medicines and 2.6% on psychiatric drugs.

### 3.2. Prevalence and Putative Risk Factors of Sexual Inactivity

The prevalence of sexual inactivity in our study population was 47.6%. The prevalence starts to increase from the 31–40 years old age group. The highest prevalence was observed in the age group >50 years old (Table 1). Table 2 shows the analysis of the association between demographic, methadone treatment factors and clinical variables, and sexual activity. The age, ethnicity, marital status, employment status, and levels of education are the significant determinants for sexual activity in univariate analysis. However, increasing age and being single or divorced are the only significant determinants for sexual inactivity in multiple logistic regression analysis (Table 3) after controlling for confounding factors. Younger age and being employed are associated with sexual activity in the single and divorced subgroups (Table 4). In the married subgroup, younger age is associated with sexual activity (Table 4). No comorbidities or medications were associated with sexual inactivity. We did not find any significant association between methadone treatment factors (dose and duration) and sexual inactivity. 

## 4. Discussion

The prevalence of sexual inactivity in this study is higher than in other populations. The prevalence of male sexual inactivity in the general population in the US and Britain are 15.2% and 15.9%, respectively [10,20]. The prevalence reported in patients receiving opioid replacement treatment by Lugoboni et al. [18] was comparable to the general population, but was four times lower than found in our study. This difference in prevalence might be due to the difference in terms of age. The mean age of our study population who were sexually inactive (51.2 + 9.3 years) was higher than their study (42.9 + 11.3 years). Moreover, the difference in study population characteristics may explain the difference between studies. Our study population was homogenous, only including MMT patients. In contrast, Lugoboni et al.’s [18] study population was heterogenous, including both MMT and BMT patients. 

Age is one of the determinants of sexual activity. Our result was similar to those found by Lugoboni et al. [18], who found that older age was significantly associated with sexual inactivity. Several studies in other populations reported similar findings [14,20]. Increasing age was also the predictive factor for sexual inactivity in both married and single/divorced subgroups.

Sexual activity in married patients was significantly higher than those who were single or divorced. Similarly, a study conducted in the Malaysian elderly general population, who were not on MMT, reported being married as one of the positive predictive factors for sexual activity with their sleeping partner [21]. Carey et al. [14] also reported a similar finding among the outpatient psychiatry population with depression, schizophrenia, and other psychiatry conditions. Low sexual activity in single/divorced people may be explained in a cultural context which forbids premarital sex [22]. The hesitancy of patients to reveal their sexual activity, particularly in single patients, is possible, as premarital sex is considered taboo in our Malaysian population. In terms of psychological context, being single or divorced are predictive factors for loneliness and depressive symptoms that may cause cessation of sexual activity [23]. However, this element was not explored in our study.

Employment status was found to be associated with sexual activity in single/divorced patients. Patients who were employed were more likely to have sexual activity than single or divorced patients. Ueda and Mercer [10] also reported similar findings in the general British population. The reason for this was explored in this study. We speculate that being employed increases the tendency to engage in a sexual relationship with a partner, as financial stability may allow person to meet social needs like dating. A study conducted in the general population in the US reported that people with a household contribution of less than 20% had more than two times the risk of being sexually inactive [20]. Lack of a sexual interest is a possible contributing factor to reduced sexual activity among unemployed persons [24]. Another possible reason for sexual inactivity for single/divorced individuals is partner preference. Fales et al. [25] reported that stable and high income are the primary mate preference for women. Other than that, we speculate that MMT patients may have a lack of confidence due to the social stigma related to opioid dependence status. Partner rejection due to social stigma and fear of sexual-transmitted disease commonly associated with opioid-dependent patients, such as HIV and hepatitis C, may also contribute to sexual inactivity.

Despite the high proportion of sexually inactive patients in our study, we failed to find an association between sexual inactivity and methadone treatment factors such as dose and duration. However, this result does not explain the causal relationship between methadone treatment factors and sexual inactivity, as the nature of the study was cross-sectional. Other mechanisms, such as inter-individual variation in terms of genetic polymorphism, which affects pharmacokinetic and pharmacodynamics of methadone, may be attributed to sexual inactivity in the MMT population. We did not find that having comorbidities such as hypertension or diabetes, or being on certain medications, were associated with sexual inactivity.

There are some limitations to our study. The study is only able to find association and not temporal relationships, as the design was cross-sectional. The study was conducted in primary health care clinics located in the urban area, may not necessarily represent rural or suburban populations. The convenient sampling technique used might introduce selection bias. The assessment of sexual activity was limited to activity with sexual partners. We did not assess other sexual acts that do not involve partners, such as masturbation. Even though psychiatric history was obtained from both patients and medical records, we might not be able to detect undiagnosed depression, as we did not screen our patients. The reason for sexual inactivity is not explored. Another limitation is no hormonal level measurement to correlate with the possible etiological cause of sexual inactivity. To our knowledge, no earlier study had explored the determinants of sexual inactivity with a focus on the MMT population. This study provides insight regarding the high prevalence of sexual inactivity and the need to explore the reasons for this condition. Sexual function is a complex concept that is not only limited to the biological aspect but includes psychological and sociocultural aspects [26]. The present study indicates that biological (age) and sociocultural aspects (marital status and employment status) are essential determinants in the MMT population. The direction of future studies should include: (1) assessment of the temporal relationship between sexual activity and potential risk factors in a longitudinal study; (2) exploration of the reason of sexual inactivity in qualitative and quantitative studies; and (3) exploration of other correlates for sexual inactivity in the MMT population in the context of a biopsychosocial approach. We recommend routine assessment in sexual activity and function [27] in the MMT population and finding the putative risk factors, so that it can be managed appropriately.

## 5. Conclusions

Sexual inactivity is common in the MMT population. The putative risk factors are related to biological and sociocultural factors, such as increasing age, being single or divorced, and unemployment. Having certain comorbidities or being on certain medications are not correlates for sexual inactivity. Routine assessment of sexual problems is essential, and proper management should be given to MMT patients. 

## Figures and Tables

**Table 1:** The age-specific prevalence of sexual activity in the sample population (*n* = 271).

Prevalence	Sexual Activity	
Overall prevalence, *n* (%)	Yes 142 (52.4)	No 129 (47.6)
Age-specific prevalence, *n* (%)		
21–30 years	6 (66.7)	3 (33.3)
31–40 years	39 (73.6)	14 (26.4)
41–50 years	47 (56.6)	36 (43.4)
>50 years	50 (39.7)	76 (60.3)

**Table 2:** Bivariate analysis of the associations between sociodemographic, methadone treatment (dose and duration), and clinical variables with sexual activity among patients on methadone maintenance treatment (MMT) (*n* = 271).

Characteristics	Sexual Activity		Crude Odds Ratio with 95% CI	t, Z, |*x*^2^ Statistics	*p*-Value
	Yes (*n* = 154)	No (*n* = 146)			
Age (years), mean (SD)	46.6 (9.7)	51.2 (9.3)	0.95 (0.92, 0.97)	−4.05	<0.001
Methadone dose (mg), mean (SD)	59.8 (30.7)	59.6 (34.6)	1.00 (0.99, 1.01)	0.06	0.950
Methadone duration (months), median (IQR)	39.5 (18.9–63.1)	29.5 (14.0–62.3)	1.01 (1.00, 1.01)	−1.67	0.094
Ethnicity, *n* (%)					0.009
Malay	107 (57.5)	79 (42.5)	1.00 (Reference)	9.47	
Chinese	22 (35.5)	40 (64.5)	0.41 (0.22, 0.74)		
Indian	13 (59.1)	9 (40.9)	1.07 (0.43, 2.62)		0.003 0.888
Other *		1			
Marital status, *n* (%)					<0.001
Single	23 (20.4)	90 (79.6)	1.00 (Reference)	118.39	
Married	110 (88.0)	15 (12.0)	28.70 (14.14, 58.23)		
Divorced	9 (27.3)	24 (72.7)	1.47 (0.60, 3.58)		<0.001 0.400
Employment status, *n* (%)					
Unemployed	28 (34.1)	54 (65.9)	1.00 (Reference)	16.07	< 0.001
Employed	114 (60.6)	74 (39.4)	2.97 (1.73, 5.11)		
Missing *		1			
Level of education, *n* (%)					0.045
No school *	1	2			
Primary	18 (36.7)	31 (63.3)	1.00 (Reference)	6.22	
Secondary	115 (55.8)	91 (44.2)	2.18 (1.14, 4.14)		
Tertiary	8 (61.5)	5 (38.5)	2.76 (0.78, 9.71)		0.018 0.115
Hepatitis B, *n* (%)					
Yes	3 (33.3)	6 (66.7)	1.00 (Reference)	NA	0.316 ^#^
No	139 (53.3)	122 (46.7)	2.28 (0.56, 9.31)		
Missing *		1			
Hepatitis C, *n* (%)					
Yes	66 (47.1)	74 (52.9)	1.00 (Reference)	3.46	0.063
No	76 (58.5)	54 (41.5)	1.58 (0.98, 2.55)		
Missing *		1			
HIV, *n* (%)					
Yes	8 (50.0)	8 (50.0)	1.00 (Reference)	0.04	0.843
No	134 (52.5)	121 (47.5)	1.11 (0.40, 3.04)		
Hypertension, *n* (%)					
Yes	24 (53.3)	21 (46.7)	1.00 (Reference)	0.02	0.891
No	118 (52.2)	108 (47.8)	0.96 (0.50, 1.82)		
Diabetes mellitus, *n* (%)					
Yes	6 (50.0)	6 (50.0)	1.00 (Reference)	0.03	0.865
No	136 (52.5)	123 (47.5)	1.11 (0.35, 3.52)		
Psychiatry disorder, *n* (%)					
Yes	4 (36.4)	7 (63.6)	1.00 (Reference)	1.18	0.277
No	138 (53.1)	122 (46.9)	1.98 (0.57, 6.93)		
Hepatitis medication, *n* (%)					
Yes	3 (75.0)	1 (25.0)	1.00 (Reference)	NA	0.624 ^#^
No	139 (52.3)	127 (47.7)	0.36 (0.04, 3.55)		
Missing *		1			
HAART, *n* (%)					
Yes	5 (45.5)	6 (54.5)	1.00 (Reference)	0.22	0.638
No	137 (52.7)	123 (47.3)	1.34 (0.40, 4.49)		
Anti-hypertensive, *n* (%)					
Yes	21 (55.3)	17 (44.7)	1.00 (Reference)	0.15	0.703
No	121 (51.9)	112 (48.1)	0.87 (0.44, 1.74)		
Diabetic medications, *n* (%)					
Yes	6 (66.7)	3 (33.3)	1.00 (Reference)	NA	0.505 ^#^
No	136 (51.9)	126 (48.1)	0.54 (0.13, 2.20)		
Psychiatry medication, *n* (%)					
Yes	3 (42.9)	4 (57.1)	1.00 (Reference)	NA	0.711 ^#^
No	139 (52.9)	124 (47.1)	1.49 (0.33, 6.81)		
Missing *		1			

Independent *t*-test was used to compare mean, Mann-Whitney test was used to compare median, the chi-squared test was used to compare categorical variables. t statistic derived from the independent *t*-test, Z statistic derived from the Mann-Whitney test and |*x*^2^ statistic derived from the Chi-squared test. *Analysis was not done due to the small number. ^#^ Fisher Exact test. CI = confidence interval; IQR = interquartile range; HAART = highly active antiretroviral therapy; HIV = human immunodeficiency; MMT = methadone maintenance treatment; NA = not applicable; SD = standard deviation.

**Table 3:** Multiple logistic regression analysis of the variables associated with sexual activity among the patients on MMT (*n* = 265).

Variables	Adjusted Odds Ratio	95% Confidence Interval	*p*-Value
Age of respondents	0.94	0.90–0.98	0.004
Marital Status			<0.001
Single	1.00	Reference	
Married	34.07	15.05–77.12	<0.001
Divorced	2.18	0.79–5.99	0.130

The model was adjusted for the age of respondents, methadone duration, ethnicity, marital status, employment, education level, and hepatitis C.

**Table 4:** Multiple logistic regression analysis of the variables associated with sexual activity among the patients on MMT and single/divorced subgroup (*n* =142) or married subgroup (*n* = 124).

Variables	Adjusted Odds Ratio	95% Confidence Interval	*p*-Value
Single/divorced subgroup ^a^			
Age of respondents	0.94	0.89–0.99	0.017
Employment Status			
Unemployed	1.00	Reference	0.044
Employed	2.92	1.03–8.30	
Married subgroup ^a^			
Age of respondents	0.90	0.83–0.98	0.019

^a^ This model is adjusted for age, methadone duration, ethnicity, employment, and hepatitis C.
